# Risk factors of earthquake inpatient death: a case control study

**DOI:** 10.1186/cc7729

**Published:** 2009-02-27

**Authors:** Jin Wen, Ying Kang Shi, You Ping Li, Li Wang, Lan Cheng, Zhan Gao, Ling Li

**Affiliations:** 1 The Chinese Evidence-Based Medicine Center & Department of Clinical Epidemiology, West China Hospital, Sichuan University, Chengdu 610041, PR China; 2 Department of Thoracic & Cardiovascular Surgery, West China Hospital, Sichuan University, Chengdu 610041, PR China

## Abstract

**Introduction:**

At 2:28 p.m. on 12 May, 2008, a devastating earthquake measuring 8.0 on the Richter scale hit Wenchuan County, Sichuan Province in southwest China, and resulted in the deaths of thousands of people. To date, few epidemiological studies have been conducted on the determinants of the mortality of patients hospitalised after an earthquake. This paper is aimed at identifying the contributing factors of mortality and providing a clinical reference for the management of those injured in earthquakes.

**Methods:**

A hospital-based case-control study was conducted. Cases included all deaths (n = 36) due to earthquake injuries in the West China Hospital. Controls were the quake survivors from the same hospital by at a ratio of four survivors to one death matched for sex and age. Data sources included death certificates and medical records. A conditional logistic regression was performed to assess the odds ratio (OR) of variables used in the study. A chi-squared test for trend was performed to reveal the possible relations between risk factor (variable) number and case fatality.

**Results:**

People with a severe traumatic brain injury (TBI) had the greatest risk of death (adjusted OR = 253.3, 95% confidence interval (CI) = 8.9 to 7208.6), followed by patients with multiple system organ failure (MSOF; adjusted OR = 87.8, 95% CI = 3.9 to 1928.3). Prior major disease and infection significantly increased the risk of earthquake-related death (adjusted OR = 14.9, 95% CI = 1.9 to 119.0; adjusted OR = 13.7, 95% CI = 1.8 to 103.7; respectively). The number of fatal cases increased as the risk factor numbers also increased.

**Conclusions:**

Severe TBI, infection, MSOF and prior major disease are the significant determinants of earthquake-related inpatient death in the 2008 Wenchuan earthquake. Future research with a large sample size including macro- and micro-level factors is needed.

## Introduction

At 2:28 p.m. on 12 May, 2008, a devastating earthquake hit Wenchuan County in southwest China's Sichuan Province. Measuring 8.0 on the Richter scale, the earthquake had a maximum intensity of φ (Modified Mercalli Intensity Scale) and a depth of 33 km. It led to 69,197 deaths, 374,176 injuries and 18,222 people being reported missing by 12:00 noon on 20 July. The severity and scale of this disaster has rarely been seen before, either in China or internationally.

The earthquake occurred in a rural and mountainous region in western Sichuan Province, about 50 miles (80 km) west-northwest of Chengdu (the location of the West China Hospital). West China Hospital of Sichuan University, as the only large-scale, state-level and general teaching hospital in the earthquake-affected area, received the largest number of injured people, treating a total of 2728 cases (872 were treated in the emergency department and 1856 were admitted) [1] from the disaster area. Until 11 October, only 36 of the injured had died in the hospital (fatality rate of 1.32%).

Understanding risk factors related to earthquake inpatient death is essential for effective patient management. Some previous studies have exposed the factors affecting casualties [2-4] and the treatment of disaster trauma [5]. Few epidemiological studies, however, have been conducted on the determinants of mortality of patients hospitalised after an earthquake. This paper is aimed at identifying the contributing factors to mortality and providing future clinical reference for management of earthquake-related injuries.

## Materials and methods

The Institutional Review Board (IRB) of the West China Hospital in Sichuan University approved this study. No informed consent was necessary as this study used existing data, which was obtained with IRB approval and informed consent from IRB.

### Study design

A hospital-based case-control study was conducted. Cases included all deaths (n = 36) in West China Hospital due to earthquake injuries for the five months after the date of the earthquake. Controls were selected from quake-related and hospitalised survivors (i.e. cases flagged on admission) in a ratio of four survivors to death matched for sex and age. Thus, the number of controls was 144. Data sources include death certificates and medical records.

### Key variables

Using the information from patient medical records, we collected data on 13 dichotomous variables: local hospital treatment; severe traumatic brain injury (TBI); crush syndrome; acute renal failure (ARF); thoracic organ injury; celiac organ injury; amputation; cranium fracture; trunk fracture; extremity fracture; infection; multiple system organ failure (MSOF); and prior major disease.

We define the above variables as follows. Local hospital treatment means the earthquake patient received primary treatment before being transferred to the West China Hospital. Severe TBI was judged as damage to the brain resulting from external mechanical force, along with a Glasgow Coma Score of 8 or below [6,7]. Crush syndrome refers to a serious medical condition characterised by major shock and renal failure following a crushing injury to skeletal muscle [8]. ARF is sudden and often temporary loss of kidney function, and was determined by physicians according to published criteria [9]. Thoracic and celiac organ injury was judged by computed tomography (CT) scan or magnetic resonance imaging (MRI), along with clinical symptoms. Infection was diagnosed by laboratory processing of patient samples, which consisted of smears for secretion substance and samples for culture. Multiplesystem organ failure was defined as a progressive condition characterised by combined failure of several organs such as the lungs, liver and kidney, along with some clotting mechanisms. Prior major disease refers to one or more of the following four types of illnesses before the shock: basal dysbolism disease, e.g. diabetes; tumour; cardiovascular diseases, e.g. hypertension; and chronic organ dysfunction, e.g. chronic renal failure.

### Statistical analysis

A conditional logistic regression was conducted to assess the odds ratio (OR) of the variables used in the case-control study. To determine the precision of the estimates, 95% confidence intervals (CI) of ORs were calculated. To reveal the possible relation between risk factor (variable) number and case fatality, a chi-squared test for trend was performed. This analytic process was performed using Stata software (Version 10, StataCorp, College Station, TX, USA).

## Results

The male to female ratio for both groups ws 1 to 1.1. The mean (standard deviation (SD)) age and days before admission were 65.8 (21.0) years and 7.3 (5.9) days for cases, and 64.3 (20.9) years and 7.2 (6.0) days for controls. Less than 20% of patients were transferred to the West China Hospital within 72 hours after the earthquake for both cases and controls.

Table 1 presents the frequency of factors used in the case-control study. The case group contained a greater percentage of severe TBI, crush syndrome, ARF, thoracic organ injury, celiac organ injury, amputation, cranium fracture, infection, MSOF and prior major disease than the control group, although the control group constituted a large proportion of local hospital treatment, trunk fracture and extremity fracture.

**Table 1:** Frequency of factors used in the case-control study, West China Hospital, China, 2008

	Cases (n = 36)		Controls (n = 144)	
		
	No.	%	No.	%
Local hospital treatment				
*No*	20	55.6	50	34.7
*Yes*	16	44.4	94	65.3
Severe TBI				
*No*	26	72.2	137	95.1
*Yes*	10	27.8	7	4.9
Crush syndrome				
*No*	31	86.1	141	97.9
*Yes*	5	13.9	3	2.1
ARF				
*No*	28	77.8	139	96.5
*Yes*	8	22.2	5	3.5
Thoracic organ injury				
*No*	27	75.0	127	88.2
*Yes*	9	25.0	17	11.8
Celiac organ injury				
*No*	30	83.3	139	96.5
*Yes*	6	16.7	5	3.5
Amputation				
*No*	32	88.9	134	93.1
*Yes*	4	11.1	10	6.9
Cranium fracture				
*No*	33	91.7	136	94.4
*Yes*	3	8.3	8	5.6
Trunk fracture				
*No*	26	72.2	92	63.9
*Yes*	10	27.8	52	36.1
Extremity fracture				
*No*	28	77.8	63	43.8
*Yes*	8	22.2	81	56.3
Infection				
*No*	17	47.2	116	80.6
*Yes*	19	52.8	28	19.4
MSOF				
*No*	24	66.7	143	99.3
*Yes*	12	33.3	1	0.7
Prior major disease				
*No*	20	55.6	118	81.9
*Yes*	16	44.4	26	18.1

ARF = acute renal failure; MSOF = multiple system organ failure; TBI = traumatic brain injury.

Table 2 lists both univariate and multivariate effects of included variables. After entering all co-variables simultaneously in conditional logistic regression analysis, severe TBI, infection, MSOF and prior major disease were the significant determinants of earthquake-related inpatient death in the 2008 Wenchuan earthquake. Patients with a severe TBI had the greatest risk of death (adjusted OR = 253.3, 95% CI = 8.9 to 7208.6), followed by patients with MSOF (adjusted OR = 87.8, 95% CI = 3.9 to 1928.3). Having a prior major disease or being infected significantly increased the risks of earthquake-related death (adjusted OR = 14.9, 95% CI = 1.9 to 119.0; adjusted OR = 13.7, 95% CI = 1.8 to 103.7; respectively).

**Table 2:** Results of conditional logistic regression analyses*, West China Hospital, China, 2008

	Univariate estimate			Adjusted estimate†		
		
	OR	95% CI	p	OR	95% CI	p
Local hospital treatment						
*No*	Ref.			Ref.		
*Yes*	0.4	0.2 to 0.9	0.025	0.2	0.0 to 1.01	0.053
Severe TBI						
*No*	Ref.			Ref.		
*Yes*	14.9	3.2 to 69.9	0.001	253.3	8.9 to 7208.6	0.001
Crush syndrome						
*No*	Ref.			Ref.		
*Yes*	6.7	1.6 to 27.9	0.009	5.5	0.2 to 139.5	0.298
ARF						
*No*	Ref.			Ref.		
*Yes*	6.4	2.1 to 19.5	0.001	2.2	0.1 to 40.0	0.589
Thoracic organ injury						
*No*	Ref.			Ref.		
*Yes*	2.4	0.9 to 5.8	0.054	0.4	0.0 to 3.9	0.397
Celiac organ injury						
*No*	Ref.			Ref.		
*Yes*	4.8	1.5 to 15.7	0.010	12.2	0.5 to 311.9	0.130
Amputation						
*No*	Ref.			Ref.		
Yes	1.6	0.5 to 5.3	0.419	0.9	0.1 to 7.9	1.000
Cranium fracture						
*No*	Ref.			Ref.		
*Yes*	1.6	0.4 to 6.5	0.530	0.4	0.0 to 9.9	0.584
Trunk fracture						
*No*	Ref.			Ref.		
*Yes*	0.7	0.3 to 1.5	0.359	1.1	0.1 to 8.5	0.896
Extremity fracture						
*No*	Ref.			Ref.		
*Yes*	0.2	0.1 to 0.5	0.001	2.8	0.5 to 14.2	0.218
Infection						
*No*	Ref.			Ref.		
*Yes*	5.2	2.2 to 12.3	0.000	13.7	1.8 to 103.7	0.011
MSOF						
*No*	Ref.			Ref.		
*Yes*	47.9	6.2 to 369.1	0.000	87.8	3.9 to 1928.3	0.005
Prior major disease						
*No*	Ref.			Ref.		
Yes	4.5	1.8 to 11.0	0.001	14.9	1.9 to 119.0	0.011

* Variables were matched for age and sex.

† Estimates are from a multivariate conditional logistic regression model (simultaneous entry of all variables) that included: local hospital treatment; severe traumatic brain injury (TBI); crush syndrome; acute renal failure (ARF); thoracic organ injury; celiac organ injury; amputation; cranium fracture; trunk fracture; extremity fracture; infection; multiple system organ failure (MSOF); and prior major disease.

CI = confidence interval; OR = odds ratio.

In univariate analysis, local hospital treatment lowered the risk of earthquake-related death (OR = 0.4, 95% CI = 0.2 to 0.9). Patients with crush syndrome, ARF and celiac organ injury had higher risk of death (OR = 6.7, 95% CI = 1.6 to 27.9; OR = 6.4, 95% CI = 2.1 to 19.5; OR = 4.8, 95% CI = 1.5 to 15.7, respectively). After adjustment for other variables, however, all four variables demonstrated no statistical significance.

Table 3 presents the case and control numbers by different risk factor numbers, along with case fatality. The chi-squared test for trend demonstrated that the case fatality increased as the factor numbers raised (*x*^2 ^= 21.864, p = 0.000).

**Table 3:** Case fatality by factor number (variable number)

Factor number*	Cases (n)	Controls (n)	Case fatality† (%)
0	0	11	0.00
1	6	60	9.09
2	5	49	9.26
3	12	14	46.15
4	9	7	56.25
≥ 5	4	3	57.14

* The variable 'local hospital treatment' was excluded, and the total number of study factors (variables) was 12.

† The chi-squared test for trend, with 1 degree of freedom, is *x*^2 ^= 21.864, p = 0.000.

## Discussion

The magnitude 8.0 earthquake that struck Sichuan Province on 12 May, 2008, was the strongest earthquake China has experienced in over 50 years. It caused the largest loss of life since the Tangshan earthquake in 1976. Identification of determinants for earthquake-related death is critical to triage, transfer, nursing and treatment, as well as for treating patients in shock. This paper revealed that severe TBI, infection, MSOF and prior major disease were the significant risk factors associated with earthquake-related inpatient death in the 2008 Wenchuan earthquake.

Over 60% of the dead were older than 60 years of age. Similar findings were published elsewhere [4,10-13]. Due to the fact that the epicentre was located in a mountainous region and there were lots of collapsed roads, the transfer of thousands of severely injured patients was delayed, which is the main reason why many patients were admitted to our hospital up to seven days after the earthquake.

People with severe TBI have a high risk of death. Bouras and colleagues [14] reported that the severe TBI case fatality rate among the senior population (65 years or older) was 86.9%, similar to the findings of a recent review [6]. This study demonstrated that earthquake inpatients with severe TBI have a large risk of death (adjusted OR = 253.3). This raises ethical questions of whether active treatment should be initiated once the severe TBI has been diagnosed, particularly among the older population. Infection, usually not a threatening factor, was a primary cause of earthquake inpatient death in our study, which means physicians need to pay more attention to testing, preventing and treating inpatients that might have an infection. Moreover, this finding was consistent with a previous study that revealed that the mortality rate was significantly higher in those patients with hospital-acquired infection than those without [15]. That the patient infection rate was notable in our hospital was high-probably related to: delayed transfer because of broken roads; heavy damage to the front-line hospital; and insufficient drugs, equipment and physicians. Although two articles [15,16] have shown that MSOF is a primary cause of death, it was the conclusion of descriptive and qualitative studies. We presented the quantitative risk of MSOF to cause death using multivariable analysis, and demonstrated that it is responsible for inpatient mortality. Notably, MSOF may or may not result from infection. An previous article [17] reported that MSOF is primarily due to infection and the most common fatal expression of uncontrolled infection. If this was also true in our study, the OR estimates might be inaccurate. However, it is very difficult to determine the causality between MSOF and infection, and both are important predictors of death. Few articles have addressed the impact of prior major diseases or original diseases on the prognosis of earthquake traumatised patients. Multivariable analysis, however, revealed that the patient's prior major disease before the earthquake is responsible for the risk of death after the earthquake. Thus, physicians must pay attention to treating the prior diseases when treating new trauma in earthquake patient.

Univariate analysis revealed that local hospital treatment, crush syndrome, ARF and celiac organ injury were related to patient death. Although the four factors failed to be recognised as influential to mortality in multivariate analysis, they should be carefully managed when considering their clinical importance. The fact that local hospital treatment might reduce the risk of death suggests that rapid medical rescue plays an essential role in saving lives. One drawback of this study is that the information of patients' staying in a local hospital before transferring to our hospital was not available. It should be remembered that delay to definitive care is one of the most important predictors of outcome after trauma. Two previous papers [13,16] reported that crush syndrome, ARF and vital organ injury account for mortality to a certain extent, similar to our findings.

Whatever the main risk factors for earthquake inpatient death are, the fact that those multi-factors (except local hospital treatment) co-existed might indeed increase the risk of patient death. The result of chi-squared tests for trend provided meaningful evidence that patients who were recorded with more risk factors were more vulnerable to death, which will benefit the earthquake patient management.

To the best of our knowledge, this paper is the first hospital-based case-control study on risk factors pertaining to earthquake inpatient death; however, there are some limitations to this study. To begin with, we failed to analyse the impact of age and sex on death because of matching. Secondly, we did not calculate sample size because this was an emergent natural disaster study, and nobody knows how many injured people will die in the hospital. This might lead to insufficient statistical power to disclose risk factors. Thirdly, the authors failed to consider the seismic and structural factors, along with time under the rubble, which could be associated with earthquake-related death. Nevertheless, compared with population-based case-control studies, this paper has provided information which directly relates to medical relief and earthquake inpatient management. Finally, a selection bias (e.g. admission rate bias or Berkson bias) should be noted. One of the most important exclusions from hospital-series case-control studies are at-scene deaths. As most earthquake deaths do occur outside the hospital, it is a critical exclusion for trauma death numbers. Therefore, the causes of death for hospitalised patients may dramatically differ from at-scene deaths. Moreover, although the discharged earthquake patients were at low risk of death, we failed to follow up with them and the post-discharge deaths (if any) were excluded. This might restrict the general applicability of our study results. However, what this paper demonstrated can be used when managing earthquake inpatients.

Based on the results of this case-control study along with the treatment experience in our hospital, some suggestions might be helpful when managing future earthquake patients as follows: older patients with earthquake trauma (especially those with prior major diseases) are the population at highest risk and more attention should be paid to them; an evidence-based system of triage should be drawn up to distinguish patients with different severities of injury; screening, treating and following up infections are vital to the patients' survival and once patients are infected, they should be isolated; and a team of multidisciplinary doctors should be organised as soon as possible to monitor, diagnose and treat critical cases, especially those in the intensive care unit.

The cause of earthquake-related death is multi-factoral and complicated. Better identification of the risk factors for death and their relation with each other can help medical providers identify vulnerable populations and take corresponding measures to reduce mortality. Future research with a large sample size, including macro- and micro-level factors, is needed.

## Conclusion

Severe TBI, infection, MSOF and prior major disease were the significant risk factors associated with earthquake-related inpatient death, and should be given more attention when managing patients after an earthquake.

## Key messages

• Understanding risk factors related to earthquake inpatient death is essential for effective patient management.

• Few epidemiological studies have been conducted on the determinants of mortality of patient hospitalised after an earthquake.

• This case-control study revealed that severe TBI, infection, MSOF and prior major disease were the significant risk factors associated with earthquake-related inpatient death.

## Abbreviations

ARF: acute renal failure; CI: confidence interval; CT: computed tomography; IRB: Institutional Review Board; MRI: magnetic resonance imaging; MSOF: multiple system organ failure; OR: odds ratio; SD: standard deviation; TBI: traumatic brain injury.

## Competing interests

The authors declare that they have no competing interests.

## Authors' contributions

WJ, SY and LY participated in the study design, data analysis and interpretation. WJ drafted the manuscript. SY is the guarantor and critically revised the manuscript. WJ, CL, GZ and LL performed the data abstraction from medical records and interpretation. WL participated in data analysis, interpretation and critical revisions of the manuscript. All authors read and approved the final manuscript.
